# Immunometabolic Regulations Mediated by Coinhibitory Receptors and Their Impact on T Cell Immune Responses

**DOI:** 10.3389/fimmu.2017.00330

**Published:** 2017-04-11

**Authors:** Nikolaos Patsoukis, Jessica D. Weaver, Laura Strauss, Christoph Herbel, Pankaj Seth, Vassiliki A. Boussiotis

**Affiliations:** ^1^Division of Hematology-Oncology, Harvard Medical School, Boston, MA, USA; ^2^Department of Medicine, Beth Israel Deaconess Medical Center, Harvard Medical School, Boston, MA, USA; ^3^Division of Interdisciplinary Medicine and Biotechnology, Beth Israel Deaconess Medical Center, Harvard Medical School, Boston, MA, USA; ^4^Beth Israel Deaconess Cancer Center, Harvard Medical School, Boston, MA, USA

**Keywords:** T cells, costimulation, coinhibitory molecules, metabolism and bioenergetics, T cell differentiation

## Abstract

Host immunity provides wide spectrum protection that serves to eradicate pathogens and cancer cells, while maintaining self-tolerance and immunological homeostasis. Ligation of the T cell receptor (TCR) by antigen activates signaling pathways that coordinately induce aerobic glycolysis, mitochondrial activity, anabolic metabolism, and T effector cell differentiation. Activation of PI3K, Akt, and mTOR triggers the switch to anabolic metabolism by inducing transcription factors such as Myc and HIF1, and the glucose transporter Glut1, which is pivotal for the increase of glucose uptake after T cell activation. Activation of MAPK signaling is required for glucose and glutamine utilization, whereas activation of AMPK is critical for energy balance and metabolic fitness of T effector and memory cells. Coinhibitory receptors target TCR-proximal signaling and generation of second messengers. Imbalanced activation of such signaling pathways leads to diminished rates of aerobic glycolysis and impaired mitochondrial function resulting in defective anabolic metabolism and altered T cell differentiation. The coinhibitory receptors mediate distinct and synergistic effects on the activation of signaling pathways thereby modifying metabolic programs of activated T cells and resulting in altered immune functions. Understanding and therapeutic targeting of metabolic programs impacted by coinhibitory receptors might have significant clinical implications for the treatment of chronic infections, cancer, and autoimmune diseases.

## T Cell Coinhibitory Receptors

When the T cell receptor (TCR) is engaged, tyrosine phosphorylation of the TCR-associated CD3 chains recruits kinases and scaffold proteins leading to the formation of a supramolecular complex that promotes activation of signaling cascades, generation of second messengers, and initiation of transcriptional events, which lead to T cell differentiation programs (1). These signaling pathways synergistically promote glycolysis and anabolic metabolism to support T cell clonal expansion and effector cell generation (2–4). It is now well understood that metabolic mediators function as intermediates between these signaling events and the functional outcome of T cell activation. Costimulatory receptors, engaged simultaneously with the TCR, have a major impact on signaling events and a decisive role in the differentiation program of T cells. Thus, their role in altering the immunometabolic programming mediated by TCR-mediated activation is unequivocal (5–7).

Our understanding about the functional role of costimulation has evolved from the two-signal model proposed by Lafferty and Cunningham to explain the activation of naïve T cells (8, 9). Although T cell costimulatory pathways were envisioned as stimulators of T cell responses by that model, it is now clear that both stimulatory (costimulatory) and inhibitory (coinhibitory) second signals exist and mediate their impact not only in naïve (T_N_) but also in effector (T_EFF_), memory (T_M_), and regulatory (T_REG_) T cells (10–12). These receptors are key regulators of T cell activation, tolerance, and exhaustion, and therapeutic modulation of costimulatory and coinhibitory pathways provides effective new treatment strategies in cancer, autoimmunity, infectious diseases, and allogeneic transplantation (13–15).

**Figure 1:**
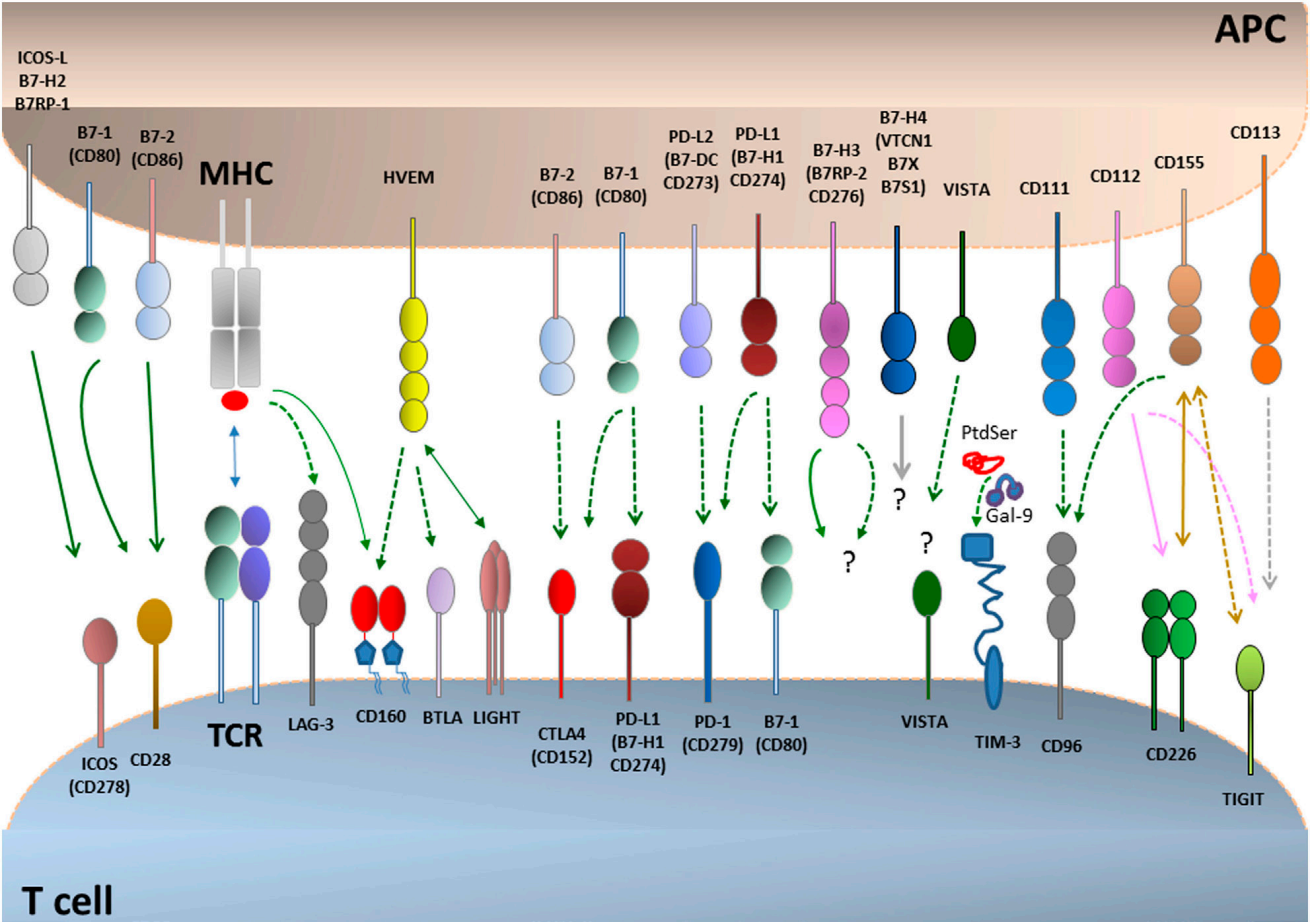
**Coinhibitory pathways**. T cell activation is initiated by recognition of antigens presented by antigen-presenting cells (APCs) to the T cell receptor (TCR)–CD3 complex in the presence of CD28 costimulation. CD28 is the prototype costimulatory receptor and interacts with CD80 and CD86. Many coinhibitory pathways are upregulated upon T cell activation and can attenuate TCR and costimulatory signals. Coinhibitory pathways in the B7–CD28 family control responses of naive, effector, regulatory, memory, and exhausted T cells. Ligands for coinhibitory receptors are expressed on APCs, non-hematopoietic cells, tumors, and some of them also on T cells. The receptors for B7-H3 and B7-H4 and their effects remain unclear. Binding ligands for VISTA have not been identified. Continuous lines indicate interactions mediating stimulatory effects, and dotted lines indicate interactions mediating inhibitory effects.

The first costimulatory receptor CD28 and the first coinhibitory receptor CTLA-4 and their shared ligands CD80 (B7-1) and CD86 (B7-2) constitute the best-characterized pathway, which serves as a paradigm for other costimulatory and coinhibitory pathways. The massive lymphoproliferation and organ infiltration by activated T cells in the CTLA-4-deficient mice (16, 17) was the first indication that coinhibitory receptors have a mandatory role in the regulation of T cell tolerance. Today, many additional costimulatory and coinhibitory receptors have been identified, and their role in the induction and maintenance of T cell tolerance has been established (Figure 1). These pathways fall into two major families: the Ig superfamily, which includes the B7–CD28, TIM, CD226–TIGIT–CD96 families as well as LAG-3, and the TNF–TNF receptor superfamily (18–20). Coinhibitory receptors provide a balance on the activation and expansion of antigen-specific T cells upon encounter with antigen but also regulate T cell tolerance by restraining the initial activation of naïve self-reactive T cells and/or responses of harmful self-reactive T cells. Coinhibitory pathways also regulate the generation and function of thymic-derived T_REG_ and T_REG_ generated at peripheral sites (21, 22). Ligands for various coinhibitory receptors are expressed on antigen-presenting cells (APCs) but also in non-hematopoietic cells (18–20). The expression of coinhibitory ligands on non-hematopoietic cells has a key role for the maintenance of tissue tolerance by suppressing the expansion and function of self-reactive T cells. Importantly, tumors and infectious agents that cause chronic infections exploit these natural tolerance mechanisms to evade surveillance and attack by the immune system (13, 23).

Coinhibitory receptors have a major impact on the differentiation fate of T cells as well as on their ability to proliferate. These functional endpoints are regulated by T cell metabolic reprogramming (24, 25). Therefore, altering metabolic reprogramming might be a key mechanism by which coinhibitory receptors modify T cell differentiation and function. Because various coinhibitory receptors mediate distinct effects on the activation of signaling pathways downstream of the TCR, the role of coinhibitory receptors on altering the metabolic programs of T cells is also anticipated to be different. Identification and targeting the specific immunometabolic pathways relayed by coinhibitory receptors might have significant clinical implications. For example, selective receptor targeting might be employed in order to achieve specific desired modifications in the metabolic programs and functional fates of T cells. In this review, first, we will discuss the current understanding of the mechanisms underlying metabolic programs induced during T cell differentiation and how these are affected by cytotoxic lymphocyte antigen-4 (CTLA-4) and programmed death-1 (PD-1), the two most clinically relevant coinhibitory receptors thus far, for which therapeutic targeting has achieved major success in cancer treatment. Next, we will review briefly other inhibitory pathways, and we will discuss lymphocyte activation gene-3 (LAG-3), T cell-immunoglobulin–mucin domain 3 (TIM-3), and T cell immunoglobulin and ITIM domain (TIGIT), which are additional promising therapeutic targets. Finally, we will briefly discuss the CD160/B and T lymphocyte attenuator (BTLA)/LIGHT/herpesvirus entry mediator (HVEM) pathway, which has the rare property to engage members of the Ig superfamily and the TNF receptor superfamily and to deliver both coinhibitory and costimulatory signals (Please see glossary in Supplementary Material).

## Immunometabolism and T Cell Differentiation

**Figure 2:**
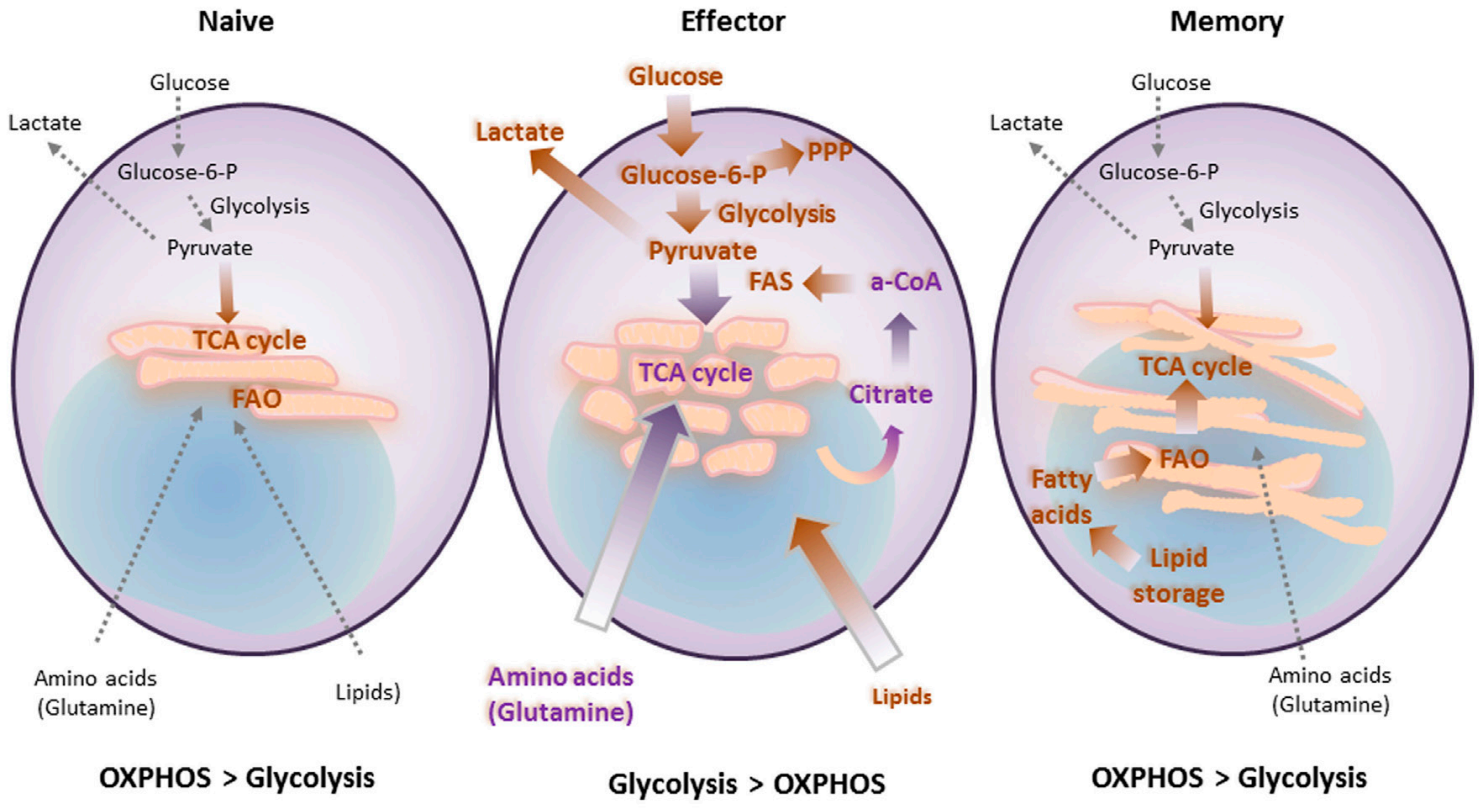
**T cell differentiation is accompanied by metabolic changes, which are affected by costimulatory and coinhibitory receptors**. Naïve T cells function in antigenic surveillance and do not proliferate. This requires minimal energetic and biosynthetic activity, which is represented by a metabolically quiescent state, and is accompanied by minimal nutrient uptake. Their only energy-demanding processes are ion homeostasis, membrane integrity, and movement. The primary ATP sources are oxidative phosphorylation (OXPHOS) and fatty acid oxidation (FAO) to fuel the low energy demand. Upon antigen encounter T cells differentiate into effector cells. This process is accompanied by metabolic changes, which are required to fulfill their new (effector) functions and rapid proliferation. Uptake of nutrients is enhanced. Glucose is the main nutrient used for energy and for generation of biosynthetic precursors. These changes combined with increased glutaminolysis and a high degree of protein, lipid, and nucleic acid synthesis support cell growth and proliferation. These metabolic changes coincide with mitochondria fission. Memory T cells do not proliferate and thus have minimal biosynthesis and nutrient uptake. However, they have increased spare respiratory capacity, which supports their ability to rapidly proliferate upon re-encounter of antigen. This cellular fate includes another metabolic adaption, which supports metabolic switch to FAO *via* increased carnitine palmitoyltransferase 1A. These metabolic and energetic changes are supported by fusion of mitochondria.

**Figure 3:**
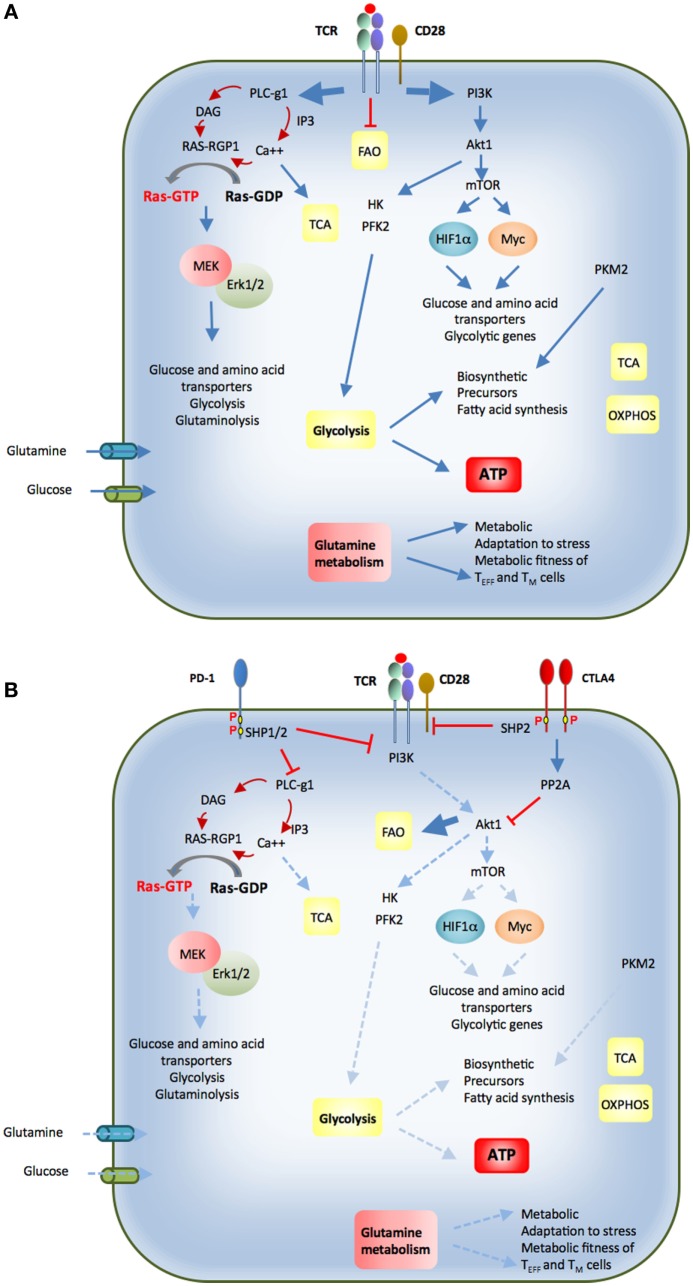
**(A)** Upon antigen encounter T cells differentiate into effector cells. Antigen binding to the T cell receptor (TCR) and coactivation by CD28 inhibit fatty acid oxidation (FAO) and activate PI3K-Akt. This activation triggers glycolytic enzymes HK and PFK2. Additionally, mTOR signaling is turned on, which enhances expression of glycolytic genes, glucose, and amino acid transporters *via* activation of transcription factors HIF1α and Myc. Activation of PLC-γ1 and generation of second messengers result in activation of Ras and MEK/Erk pathway, which is required for expression of nutrient transporters and nutrient utilization. Calcium release activates calcium-dependent mitochondrial dehydrogenases, which activate the TCA cycle. Effector T cells also switch from balanced PKM1 and PKM2 expression to increased and predominant expression of PKM2, which promotes generation of biosynthetic precursors. These events promote glucose and glutamine uptake, increased glycolysis and glutaminolysis combined with a high degree of protein, lipid, and nucleic acid synthesis to support cell growth and proliferation. CD28 costimulation is required for activation of the signaling pathways that support these metabolic changes. **(B)** Cytotoxic T lymphocyte antigen-4 (CTLA-4) and programmed death-1 (PD-1) coinhibitory receptors are expressed in activated T cells. *Via* the recruitment of phosphatases, both these coinhibitory receptors attenuate the signaling events mediated by ligation of the TCR by antigen and have a mandatory role in the metabolic changes required for optimal T cell activation, function, and differentiation. CTLA-4 opposes the effects of CD28 costimulation and can inhibit potent TCR-mediated signals. PD-1 inhibits weak but not strong TCR signals. The imbalanced activation of these signaling pathways alters the metabolic reprogramming of T cells and their differentiation fate (see text for details).

Similar to the metabolism of other non-proliferating cells, naïve T cells depend on catabolic metabolism to meet their energetic needs, relying predominantly on oxidative phosphorylation (OXPHOS) to generate ATP (26). Extrinsic IL-7 survival signal maintains naïve T cell quiescence, a mechanism that relies on tuberous sclerosis (TSC) function to keep mTOR activation in check (27–29). Upon antigen encounter, T cells switch to anabolic metabolism to meet the increased metabolic demands of biomass accumulation, proliferation, and biosynthesis of mediators of effector functions (Figures 2 and 3A). Signaling *via* the TCR is sufficient to upregulate expression of the glucose transporter Glut1 *via* Myc (30). However, the metabolic reprogramming accompanying the differentiation into T_EFF_ requires costimulation. Signaling *via* CD28 costimulation activates the PI3K/Akt/mTOR pathway, which is crucial to activate aerobic glycolysis required for T cell differentiation and function (6, 31, 32). mTOR has a key role in regulating metabolism coupling nutrient availability to cell growth and division (33, 34).

After pathogen clearance, antigen-specific T cells undergo contraction, and most T_EFF_ cells undergo cell death. A small subset survives and goes on to become T_M_ cells. T_M_ cells exhibit a distinctive increase in mitochondrial mass and higher spare respiratory capacity (SRC) (35). This is a consequence of fusion in the mitochondria of T_M_ cells, which configures associations of the electron transport chain and favors OXPHOS (36). In contrast to T_EFF_, which rely predominantly on glycolytic metabolism, T_M_ rely on mitochondrial fatty acid oxidation (FAO) of *de novo* synthesized FA, rather than uptake of lipids (37). During TCR engagement with APCs, T cells can divide asymmetrically. It has been determined that the APC-proximal daughter generated during cell division is more likely to become a T_EFF_ while the APC-distal daughter cell is more likely to differentiate into a T_M_ (38). In addition to its role in promoting glycolysis, asymmetric Myc sorting between the daughter cells plays a role in the distribution of amino acid content and transporters as well as mTORC1. This results in altered metabolism and thus altered proliferation and differentiation that may give rise to effector versus memory fates of the daughter cells (39). This is likely not the only mechanism to generate T_M_ cells, as other studies have shown that T_EFF_ cells are the ones that can give rise to T_M_ (40, 41). Enforcing the metabolic switch to FAO by enhancing AMPK or by inhibiting mTOR results in increased numbers of T_M_ cells (42, 43). In mammalian cells, mTOR forms functionally distinct complexes—mTORC1 and mTORC2. Raptor and Rictor are the core adaptor subunits of mTORC1 and mTORC2, respectively. Interestingly, T cells deficient in Rictor show enhanced CD8 memory formation with enhanced recall responses without dampening effector functions (44).

Since the activation of T cells requires accompanying metabolic reprogramming for differentiation, it is no surprise that metabolism drives the differentiation of CD4 T helper (Th) subsets as well. Th1, Th2, Th17, and T_REG_ cells are the best metabolically defined thus far. Initial evidence showing their metabolic distinction came when suppression of mTOR by rapamycin prevented T_EFF_ proliferation and instead promoted T_REG_ generation, even in Th17 polarizing conditions (45). Genetic deletion of mTOR in T cells confirmed the results of pharmacological inhibition and caused production of only T_REG_, illustrating the obligatory role of mTOR for T_EFF_ production (46). Furthermore, T_EFF_ show a strong preference for glycolytic rather than mitochondrial metabolism, whereas T_REG_ rely more on the oxidation of lipids (47).

Th17 cells are particularly dependent on glycolysis. Although Myc is dominant over the hypoxia-inducible factor 1α (HIF1α) in regulating differentiation of naïve T cells into T_EFF_ cells (30), HIF1α has a key role in the metabolic switch to aerobic glycolysis that influences the balance of Th17/T_REG_ in favor of Th17 cells. Genetic deletion of HIF1α in mice or blocking glycolysis with 2-deoxyglucose showed reduced Th17 and enhanced T_REG_ development (48). HIF1α in coordination with RORγt and p300 promotes the development of Th17 cells while concurrently weakening the development of T_REG_ cells by promoting proteasomal degradation of FoxP3 (49). Furthermore, differentiation of Th1 and Th17 cells is selectively regulated by mTORC1 while Th2 differentiation is regulated by mTORC2 (50).

It should be noted that glycolytic enzymes themselves might also have an active role in regulating T_EFF_ cell function. For example, it has been proposed that when glycolytic rates are low in Th1 CD4^+^ T cells, glyceraldehyde phosphate dehydrogenase (GAPDH) binds to and suppresses IFN-γ mRNA translation (51). In effector memory CD8^+^ T cells, glycolysis was observed to impact chromatin remodeling in the promoter region of the IFN-γ gene, thus facilitating gene transcription (52). In addition, naïve T cells express both isoforms of the glycolytic enzyme pyruvate kinase, PKM1 and PKM2, which catalyzes the conversion of phosphoenolpyruvate to pyruvate. After activation, the M2 isoform rapidly accumulates and is the dominant form expressed in T_EFF_ cells (53, 54). The M2 isoform is less efficient, but this skews glycolysis toward biosynthetic pathways and may give cells a growth advantage during rapid proliferation (55). Because coinhibitory receptors are induced after T cell activation, a process that is dependent on glycolysis, there might be a correlation between the expression and function of glycolytic enzymes and coinhibitory receptors. These findings provide evidence for an intimate causative link among TCR-mediated signaling, T cell metabolism, and T cell differentiation.

## TCR-Mediated Signaling Events Drive T Cell Metabolic Reprogramming

Signaling pathways activated by engagement of the TCR and costimulatory receptors and CD28, the prototype costimulatory receptor, activates the PI3K/Akt/mTOR and MEK/Erk MAPK pathways, and their simultaneous activation is required to promote T cell growth, division, and cytokine production (56) (Figure 3A). Autocrine and paracrine cytokine signaling loops induce further PI3K and MAPK activation, together with JAK/STAT signaling (57, 58). PI3K catalyzes the production of the second messenger phosphatidylinositol 3,4,5-trisphosphate [PI(3,4,5)P3], leading to the activation of several downstream kinases, including Akt, its most prominent effector. In naïve T cells, active Akt leads to increased Glut1 surface expression, improved coupling of HKII to the mitochondria, and increased rates of glycolysis, which correlates with proliferative capacity (6, 7, 53). Akt also promotes aerobic glycolysis by direct phosphorylation and activation of glycolytic enzymes, such as PFK2 (59), by regulating Glut1 trafficking, and by inducing several transcription factors (60). Akt can activate mTORC1, a key driver of anabolic metabolism (61).

MAP kinases have a central role in metabolic regulation during T cell activation by affecting both glucose and glutamine uptake and metabolism (2, 62). One target of MAPK with a direct role on metabolic regulation is HIF1α. MAPK promotes the ability of HIF1α to activate transcription and expression of many glycolytic genes, while mediating suppression of genes involved in OXPHOS (63). HIF1α levels are also upregulated by PI(3,4,5)P3 signaling through mTOR (61) providing a mechanistic explanation for the requirement of simultaneous activation of PI3K and MAPK pathways for optimal effector T cell expansion (56). The critical role of HIF1α in T cell differentiation and function is underlined by the observation that T cells with constitutively elevated HIF1α display sustained increase of aerobic glycolysis and constitutively maintain effector function (64).

After TCR and costimulatory receptor engagement, T cell activation is maintained by sustained signaling from IL-2 and other cytokines acting on common gamma chain (γc) cytokine receptor complexes (65). This effect of IL-2 leads to glycolysis and anabolic metabolism by activating transcriptional upregulation and trafficking of Glut1. This is mediated by Jak-STAT5 signaling (66) and also by activation of PI(3,4,5)P3/Akt (57).

T cell receptor signaling pathways also lead to the generation of second messengers, diacylglycerol (DAG), and calcium. Calcium flux from intracellular stores stimulates the calcium-activated mitochondrial dehydrogenases that drive the TCA cycle (67). In addition, calcium flux downstream of the TCR causes a short-term phosphorylation of AMPK mediated by CAMKK (68). AMPK acts as an important metabolic checkpoint to control cellular anabolic pathways and to induce energy-conserving mechanisms under conditions of nutrient shortage or cellular stress. AMPK activation early during the initiation of T cell stimulation may facilitate transient increase of OXPHOS before the cells switch their metabolism toward glycolysis thereby supporting the energy demands at this early T cell activation stage. Mechanistically, AMPK activation is induced by the upstream kinase LKB1 when the cellular levels of ATP are diminished and the levels of AMP and ADP are increased in response to physiological stress (69). During T cell activation, in addition to regulation of energy, AMPK also provides metabolic fitness to the differentiating T_EFF_ and T_M_ cells (70). After activation, AMPK phosphorylates several substrates involved in the metabolism of glucose, lipid, and proteins, in autophagy, cell cycling, and cell growth (71). Activation of the TSC1/TSC2 complex by AMPK can negatively regulate the mTOR pathway (72, 73) not only by inhibiting mTOR activation *via* Rheb (74), a member of the Ras family of GTPases, but also by mediating phosphorylation of the mTORC1 partner Raptor (75).

## Coinhibitory Receptors Target Signaling Events That Drive T Cell Metabolic Reprogramming

The key signaling pathways that are activated by antigen encounter are targets of costimulatory and coinhibitory receptors (Figure 3B). Costimulatory receptors amplify TCR-mediated signaling and impact T cell metabolic reprogramming. For example, CD28-mediated costimulation mediates transcriptional upregulation of the glucose receptor Glut 1 (6) and the glutamine receptors Snat1 and Snat2 (62) after TCR ligation. Costimulation also amplifies MAPK and PI3K/Akt activation, and the simultaneous engagement of these signaling pathways is mandatory for cell cycle progression and cytokine production (56). Conversely, coinhibitory receptors oppose the signaling and functional effects induced by TCR and costimulatory pathways, thereby limiting T cell expansion and cytokine production and altering the ability of T cells to differentiate into T_EFF_ and T_M_ cells. Importantly, coinhibitory receptors do not induce global signal inhibition but rather selective targeting of signaling, resulting in imbalanced activation of pathways that are normally engaged by TCR-plus-costimulation. As a consequence, the implications of coinhibitory pathways on the metabolic and functional programs of T cells might significantly vary, dependent on the signaling targets of these inhibitory receptors.

### Cytotoxic T Lymphocyte Antigen-4

The coinhibitory receptor CTLA-4 is a central negative regulator of T cell activation. CTLA-4 is a homolog of CD28 and binds to the same ligands, CD80 (B7-1) and CD86 (B7-2), but with higher affinity (76). CTLA-4 is induced in CD4^+^ and CD8^+^ T cells after activation (77) but is constitutively expressed on T_REG_ cells, because it is a direct transcriptional target of Foxp3 (22). Crosslinking of the CTLA-4 simultaneously with TCR and CD28 inhibits cell cycle progression (78) and suppresses IL-2 production leading to proliferative arrest (79, 80). Engagement of CTLA-4 can induce an anergic phenotype similar to that observed in response to TCR stimulation alone (78) establishing the notion that CTLA-4 might function to counteract CD28-mediated costimulation. Notably, the inhibitory functions of CTLA-4 on CD4^+^ T cells appear to be more important for the prevention of autoimmunity as CTLA-4-deficient CD8^+^ T cells are incapable of inducing autoimmune pathology in the absence of CD4^+^ T cells (81, 82). This may be due to the fact that CTLA-4 expression in CD4^+^ T_REG_ is indispensable for the maintenance of T_REG_ suppressive function (22). However, direct CTLA-4-mediated inhibition of CD8^+^ T cells may be particularly important in the effector/memory CD8^+^ T cells in which CTLA-4 controls secondary responses (83, 84).

CTLA-4 controls T cell activation by selectively reversing CD28-mediated costimulation (79, 85). Because CTLA-4 is the high-affinity ligand for CD80 and CD86, CTLA-4 might inhibit activation by competing with CD28 for interaction with CD80 and CD86. It can also downregulate the expression of CD80 and CD86 expression on APCs (22) or remove these costimulatory ligands from APCs through transendocytosis and, as a consequence, reduce CD28 engagement (86). After binding to CD80/CD86, CTLA-4 may also alter the properties of dendritic cells by inducing the tryptophan-degrading enzyme IDO, a known T cell inhibitor (87).

CTLA-4 also inhibits T cell activation through cell-intrinsic signaling mechanisms (88). The cytoplasmic tail of CTLA-4 interacts with several molecules that are key components in TCR and CD28-mediated signaling pathways (Figure 3B). TCR/CD28-mediated stimulation induces activation of several kinases, including Lck, Fyn, Lyn, Rlk, and Jak2, which are capable of phosphorylating Y165 and Y182 of the CTLA-4 cytoplasmic domain (89–92). Phosphorylation at Y165 creates a docking site for the protein tyrosine phosphatase SHP-2, which subsequently inhibits proximal TCR signaling *via* dephosphorylation of the TCRζ chain, LAT, and the Ras regulator p52SHC (93, 94). CTLA-4 interferes with the formation of lipid rafts, TCR:ZAP70 microclusters, and the central supramolecular activation complex, each of which plays important roles in T cell activation (89, 95, 96). CTLA-4 has also been identified to interact with the serine/threonine phosphatase PP2A, leading to inhibition of Akt (97, 98).

The effects of CTLA-4 on depleting CD80 and CD86 costimulatory ligands and inhibiting TCR signaling pathways have major implications on regulation of metabolic reprogramming by cell-intrinsic mechanisms. By dampening CD28-mediated signaling, which has a dominant role in regulating Glut1 expression and HKII activation (5, 6), CTLA-4 directly targets a key regulator of glucose transport, affecting intracellular glucose availability and glycolysis. Furthermore, by decreasing calcium release, which is involved in the activation of the TCA cycle (67), CTLA-4 can suppress not only glucose uptake and metabolism but also oxidation of other metabolites in the TCA cycle. Furthermore, CTLA-4 inhibits production of IL-2 (79, 80), by impairing the activation of and nuclear accumulation of AP-1, NFAT, and NF-κB (99, 100). Because IL-2 serves as an autocrine mechanism that activates and amplifies T cell metabolic reprogramming toward a glycolytic phenotype (57), inhibition of IL-2 is an additional mechanism by which this coinhibitory receptor shuts off the activation of metabolism-related expansion and differentiation of T cells downstream of the TCR and CD28.

A unique feature of CTLA-4 biology is related to its intracellular localization and trafficking. Specifically, the majority of CTLA-4 resides within intracellular vesicles of the trans-Golgi network and endosomal compartments (101, 102). In resting T cells, a small amount of CTLA-4 protein cycles continuously from the Golgi apparatus to the cell surface, followed by rapid endocytosis and lysosomal degradation (103). This intracellular trafficking is mediated by the interaction of the cytoplasmic tail of CTLA-4 with the clathrin-associated adaptors AP-1 and AP-2/AP50 (104, 105). During TCR ligation, CTLA-4 protein expression is increased, and CTLA-4-containing intracellular vesicles relocalize to the immune synapse (102). The TCR-proximal kinases Lck and ZAP70 phosphorylate the cytoplasmic tail of CTLA-4 at Y165. This event leads to disruption of the CTLA-4:AP-2 association and retention of CTLA-4 at the cell surface in the immune synapse (106). The more potent the TCR-mediated stimulation, the more CTLA-4 accumulates at the immune synapse. By this mechanism, CTLA-4 provides a dynamic inhibitory signal, which is fine-tuned by the intensity of the TCR signal (107). The continuous surface recycling and endocytosis of CTLA-4 in T cells and its rapid stabilization on the cell surface after T cell activation might be responsible for the potent inhibitory effect of CTLA-4, which is mediated early upon initiation of T cell activation for maintenance of an immune quiescent phenotype. Consistent with the biochemical evidence that CTLA-4 might rapidly inhibit TCR and CD28-mediated signaling, gene expression studies indicate that the net outcome of CTLA-4 ligation is the suppression of activation transcripts downstream of the TCR without specific targeting on metabolism-related genes (108).

### Programmed Death-1

PD-1 is a transmembrane coinhibitory receptor that controls T cell activation, exhaustion, tolerance, and resolution of inflammation. PD-1 appears within 24 h of T cell activation, declines with the clearance of antigen, and reappears in TM cells (109, 110). NFATc1, IRF9, and Notch promote PD-1 transcription (111–113), providing evidence that PD-1 expression requires not only TCR-proximal signaling but also generation of second messengers. When T cells are exposed to antigen for prolonged periods of time (as with chronic infection or cancer), the level of PD-1 expression remains high, and T cells undergo various changes in transcription factor expression, which alter their differentiation program and guide the development of a distinct state termed “exhaustion.” It should be noted that this functional state is not mediated exclusively by PD-1. Under these conditions, exhausted T (T_EX_) cells express multiple other coinhibitory receptors in addition to PD-1, making them susceptible to inhibition by multiple checkpoint pathways. Depending on the number of coinhibitory receptors expressed, T_EX_ cells display a different level of functional incompetence (114, 115). PD-1 can also be expressed on T_REG_ and T follicular regulatory cells (21, 116), as well as on natural killer (NK), NKT, B cells, macrophages, and some DC subsets during immune activation and chronic inflammation (20, 110, 117).

PD-1 has two known ligands, programmed death-ligand 1 (PD-L1; also known as CD274 and B7-H1) and programmed death-ligand 2 (also known as CD273 and B7-DC) (20, 118). PD-1 ligands are expressed on APCs and other hematopoietic cells but also on non-hematopoietic cell types and are induced or upregulated by proinflammatory cytokines such as type I and type II interferons, TNF-α, VEGF, and GM-CSF. The induction of PD-1 ligands by proinflammatory signals might serve as a negative feedback mechanism that downregulates effector T cell activity, thereby protecting tissues from excessive immune damage. These mechanisms are exploited by several pathogens and tumors to avoid attack from the immune system (13, 23).

Binding of PD-1 ligands to PD-1 results in tyrosine phosphorylation of the PD-1 cytoplasmic domain and recruitment of the tyrosine phosphatase SHP-2 (119). These events correlate with attenuated signals downstream of the TCR, decreased T cell activation and impaired cytokine production (Figure 3B). Signaling through PD-1 inhibits PI3K activity (120, 121). The main negative regulator of the PI3K/Akt pathway is PTEN, which is a lipid phosphatase, dephosphorylating PI(3,4,5)P3 at the 3′ position to generate PI(4,5)P2 (122). By limiting the amount of PI(3,4,5)P3, PTEN opposes the activation of PI3K and therefore diminishes the pro-survival and proliferative signals mediated by PI3K/Akt (123). PD-1 activates PTEN phosphatase by inhibiting its inactivating phosphorylation by CK2 (120), leading to diminished activation of Akt and decreased expression of critical transcription factors such as Tbx21 (T-bet), Eomes, and Gata3, which are involved in the function of effector cells. PD-1 ligation also inhibits Ras-MEK-ERK pathway signaling, possibly *via* SHP-2-regulated dephosphorylation of PLCγ1 (124), which is required for Ras activation *via* calcium- and DAG-dependent activation of Ras-GRP1 (125). Thus, PD-1 targets many pathways that have a decisive role in metabolic reprogramming of T cells after antigen encounter.

As a consequence of these signaling events, after PD-1 engagement, T cells have significantly diminished capacity to activate glycolysis, have reduced respiration, a function that depends on mitochondria, and also have altered lipid metabolism characterized by suppression of fatty acid synthesis and elevated FAO (126). Subsequently, T cells are unable to become effectors, a fate that requires engagement in glycolysis as well as potent mitochondrial function (51). Instead, PD-1 can promote the differentiation of T_REG_ cells, which suppress T_EFF_ functions (21). Because T_REG_ differentiation depends on FAO and lack of glycolysis (47), it is possible that the effects of PD-1 on T cell metabolic reprogramming are responsible for the switch of the T cell functional fate from T_EFF_ to T_REG_ differentiation. Such effects on immunometabolic programs may also have a causative role in the control of peripheral tolerance by PD-1 because PD-1 signaling inhibits aerobic glycolysis, which is required for Th1 and Th17 cell differentiation (51) and metabolically reprograms T cells from aerobic glycolysis to FAO (126), which promotes the generation of T_REG_ cells. Notably, the downregulation of Akt, mTOR, and S6K, which is induced by PD-1, has been previously identified as a requirement for the development of peripheral T_REG_ cells (127). Moreover, PD-1 ligation in T_EFF_ cells might reprogram T_EFF_ cells from aerobic glycolysis and FAS to catabolic lipid metabolism and FAO. By selectively inducing metabolic reprogramming of T_EFF_ cells from glycolysis to FAO, PD-1 can guide differentiation of T_EFF_ cells to T_REG_ to terminate the ongoing immune responses and to T_M_ cells to maintain the T cell repertoire as differentiation of both these cell programs depends on FAO (37, 47).

By targeting metabolic reprogramming, the PD-1:PD-L1 pathway might also alter metabolism in the tumor microenvironment. Changes in metabolism of T cells and tumor cells will have a central role in antitumor immune responses. Highly glycolytic tumor cells reduce glucose levels in the tumor microenvironment, thereby reducing the ability of tumor-infiltrating lymphocytes (TILs) to mount effector antitumor immune responses. Moreover, ligation of PD-1 by PD-L1 expressed by tumor or APC of the tumor microenvironment shuts off glycolysis and mitochondrial function of TIL leading to impaired effector function. Simultaneously, by this mechanism, T_REG_ cells are preferentially generated in the tumor microenvironment leading to progressive immunosuppression through additional T_EFF_ cell-extrinsic mechanisms.

### Other Coinhibitory Pathways

In addition to CTLA-4 and PD-1, multiple other coinhibitory molecules exist including LAG-3, TIM-3, TIGIT, CD160, BTLA, B7-H3, B7-H4, VISTA, CD244, HHLA2, and BTNL2 (Figure 1). Several of these coinhibitory receptors are expressed on dysfunctional T cells similarly to PD-1. T cell subsets coexpressing multiple coinhibitory receptors are more dysfunctional than those expressing each coinhibitory receptor alone. The coexpression of coinhibitory receptors has important clinical relevance because combined blockade with PD-1 significantly enhances therapeutic benefit.

### Lymphocyte Activation Gene-3

LAG-3 is a CD4 homolog that binds MHC class II molecules with higher affinity than CD4 (128). Among T cells, LAG-3 is expressed on activated CD4^+^ and CD8^+^ T cells, as well as in thymic and peripherally induced T_REG_ (129). It is also expressed in NK cells, NKT cells, B cells, and plasmacytoid dendritic cells. LAG-3 negatively regulates T cell proliferation and homeostasis (130, 131). The KIEELE motif of the cytoplasmic domain of LAG-3 is involved in its inhibitory function (132), but the downstream signaling pathways mediating the inhibitory effect have not been identified. LAG-3 regulates peripheral T cell tolerance by controlling self-reactive effector cells and T_REG_ (133–135). Mice lacking both LAG-3 and PD-1 rapidly develop lethal, systemic autoimmunity (136), providing evidence that these two pathways control T cell tolerance in a synergistic manner.

T cells in chronic viral infections and tumors often coexpress PD-1 and LAG-3. Furthermore, consistent with the synergistic effect in T cell autoimmunity induced by combined deficiency of PD-1 and LAG-3, combined blockade of PD-1 and LAG-3 in the lymphocytic choriomeningitis virus (LCMV) chronic infection model or in tumor models has a greater therapeutic benefit than blockade of each coinhibitory receptor alone. In this regard, LAG-3 and PD-1 blockade together can induce almost complete tumor regression in mice with established MC38 or SA1N tumors, in contrast to only ~15% efficacy when only one of the two receptors is blocked (137). Similarly, in TILs from patients with ovarian cancer, simultaneous LAG-3 and PD-1 blockade restores responsiveness of NY-ESO-1-specific LAG3^+^/PD-1^+^ CD8^+^ T cells to a greater extent than blockade of each coinhibitory receptor alone. Thus, although it remains unclear how LAG-3 affects signaling pathways regulated by TCR, the functional synergy between PD-1 and LAG-3 suggests that LAG-3 might target pathways not affected by PD-1 and, as a consequence, combined signals mediated by these coinhibitory receptors result in more profound impairment of T_EFF_ cell function.

Recent studies have shown that LAG-3 is cleaved within the short connecting peptide between the membrane proximal D4 domain and the transmembrane domain, which is mediated by ADAM family metalloproteases, resulting in the release of a soluble form of LAG-3 (sLAG-3) *in vitro* and *in vivo* (138). LAG-3 expression and function might be linked to oxidative signals generated by metabolic reprogramming of activated T cells. Treatment with an antioxidant superoxide dismutase (SOD)-mimetic compound prevented type I diabetes in mouse models by inhibiting metalloprotease activity resulting in maintenance of LAG-3 surface expression at levels adequate to attenuate TCR-mediated Th1 cell activation and effector function (139). It has also been determined that LAG-3^−/−^ splenocytes exhibit enhanced SRC, which correlates with increased mitochondrial mass and expression of the mitochondrial biogenesis transcription factor TFAM and mitochondrial complexes I–IV (140). Moreover, after suboptimal stimulation with Con-A, LAG-3^−/−^ splenocytes demonstrated greater upregulation of glycolysis-associated genes together with elevated expression of Myc and mTOR signaling (140), which are crucial for driving aerobic glycolysis. Thus, LAG-3 might regulate T cell exhaustion in conjunction with PD-1 because LAG-3 and PD-1 signals converge into regulating glycolytic metabolism and mitochondrial function both of which are involved in the differentiation of TEFF cells.

### T Cell-Immunoglobulin–Mucin Domain 3

TIM-3 was initially identified at the cell surface of IFN-γ-producing CD4^+^ Th1 and CD8^+^ cytotoxic T cells (141). In addition, TIM-3 is expressed in T_REG_ cells and innate immune cells such as DCs, NK cells, and monocytes. The discovery of TIM-3 led to the identification of the TIM family of genes (142), and examination of TIM-3 function with TIM-3-deficient mice, blocking antibodies, and TIM-3-Ig fusion protein suggest that TIM-3 family members function as negative regulators of T cell responses (141, 143, 144). Several ligands for TIM-3 have been identified, including C-type lectin galectin-9, phosphatidylserine (PtdSer), HMGB1, and CEACAM-1 (145–147). The mechanism of TIM-3-mediated inhibitory function differs from that of other coinhibitory receptors because TIM-3 has the unique ability to induce PtdSer-mediated recognition and phagocytosis. It has been postulated that blocking TIM-3 alters the immune response induced by recognition of dying tumor cells (148).

TIM-3 does not have a classical signaling motif in its cytoplasmic tail but contains five conserved tyrosine residues. Y256 and Y263 can be phosphorylated by Src kinases (149) or ITK (150) and are involved in the binding of Bat3 (HLA-B associated transcript 3), p85 PI3K, Fyn, and/or Lck (149, 151). When TIM-3 is not bound to its ligands, Bat3 is bound to TIM-3 and blocks SH2 domain-binding sites in TIM-3 tail and recruits the catalytically active form of Lck that preserves and promotes T cell signaling. In contrast, in the absence of Bat3, interaction of TIM-3 tail with the catalytically inactive form of Lck pY505 increases (151). Galectin-9 and CEACAM-1 binding to TIM-3 leads to Y256 and Y263 phosphorylation and release of Bat3 from the TIM-3 tail, thus promoting TIM-3-mediated T cell inhibition by allowing binding of Src kinases, preferentially Fyn (147, 151). Interestingly, Fyn and Bat3 compete for the same region on the TIM-3 tail. Although it is clear from these studies that the cytoplasmic tail of TIM-3 has the potential to interact with multiple components of the TCR complex, the precise mechanisms *via* which TIM-3 alters T cell signaling remains to be elucidated.

It has been determined that TIM-3^+^ T cells from HIV-infected patients display defective stimulation-induced phosphorylation of Stat5, ERK1/2, and p38 but have higher basal phosphorylation of these pathways (152). These CD8^+^TIM-3^+^ dysfunctional T cells lacked PD-1 expression suggesting that TIM-3 might be an alternative exhaustion-related inhibitory receptor and biomarker. However, when coexpressed with PD-1, TIM-3 marks the most dysfunctional/exhausted population in patients with chronic viral infections (153–155). Similarly, coexpression of TIM-3 with PD-1 also marks the most dysfunctional T_EX_ CD8^+^ cells in cancer (156).

Because TIM-3 targets key metabolism-related signaling pathways, such as PI3K/Akt/mTOR (157), and is highly expressed on dysfunctional or T_EX_ cells in chronic infections (152–155, 158) and TILs in various types of cancers (159–161), it is possible that TIM-3-mediated effects alter metabolism of T effector cells in a manner similar to PD-1. Alternatively, the additive and synergistic effect of combined PD-1 and TIM-3 blockade in the functional re-invigoration of T_EX_ cells might be explained by a selective role of TIM-3 on metabolic programs that are not affected by PD-1.

### T Cell Immunoglobulin and ITIM Domain

The coinhibitory receptor TIGIT (also called Vsig9, Vstm3, or WUCAM) is an immunoglobulin superfamily member (162, 163). It belongs to a group of stimulatory and inhibitory receptors and ligands that bind to these receptors (164). TIGIT and the costimulatory receptor CD226 (DNAM-1) share the ligands CD155 and CD112 with TIGIT as their high-affinity receptor. TIGIT also binds to CD113 whereas CD155 also binds to the inhibitory receptor CD96 (Figure 1). CD155 and CD112 are expressed on APCs and on several cancers, whereas CD155 is also expressed on several non-hematopoietic cells. TIGIT is induced after T cell activation and is expressed in T_EFF_, T_M_, T_REG_, and NK cells. TIGIT can exert immunosuppressive effects by various mechanisms. By engaging CD155 on dendritic cells, TIGIT can inhibit IL-12 production by dendritic cells, thereby preventing Th1 differentiation and functional responses (165). TIGIT also can mediate cell-intrinsic inhibitory effects on T cells and can inhibit priming of CD4^+^ and CD8^+^ T cells (166). Thus, TIGIT can promote T cell tolerance by controlling both the activation of self-reactive T cells and the function of T_REG_ (167).

TIGIT cytoplasmic tail has an ITIM motif and an immunoglobulin tail tyrosine (ITT)-like motif (164). The ITT-like motif plays a critical role in inhibitory signaling as binding of CD155 to TIGIT induces phosphorylation of the ITT motif and recruitment of SHIP1, which correlates with inhibition of NF-κB pathway activation. TIGIT is expressed on CD8^+^ TILs either alone or together with PD-1 in various human cancer types (168). In mouse cancer models, in contrast to minimal effects of blockade of TIGIT or PD-L1 alone, combined blockade of TIGIT and PD-L1 can induce substantial tumor regression and improved survival by enhancing the function of CD8^+^ TILs (169). Similarly, synergistic effects of combined PD-L1 and TIGIT blockade have been observed in the LCMV chronic viral infection model to reverse T cell exhaustion and enhance viral control (169). These findings suggest functional synergies between PD-1 and TIGIT and support exploration of combined TIGIT and PD-1 blockade for re-invigoration of immune responses.

### CD160/BTLA/LIGHT/HVEM

CD160 was discovered in an effort to identify NK cell-specific receptors (170) and contains a single IgV-like domain and a glycosyl-phosphatidylinositol (GPI)-anchor (171). CD160 interacts with classical and non-classical MHC class I molecules with weak affinity (172). CD160 is expressed in secondary lymphoid organs, intestinal lymphocytes, and peripheral CD8 and NK cells (171). Although originally thought to be involved in the cytolytic activity of CD8^+^ T cells, CD160 was subsequently identified as one of the coinhibitory receptors expressed in CD8^+^ T_EX_ cells. In fact, together with PD-1, CTLA-4, LAG-3, and CD224, CD160 is one of the coinhibitory receptors that are progressively upregulated as the CD8^+^ cell effector function declines during the differentiation to T_EX_ cells (115). Increasing numbers of CD160^+^ T cells are detected during HIV infection and are higher in patients who have optimal control of HIV (173) providing, once more, evidence about the active role of T_EX_ cells in the containment of chronic viral infections.

B and T lymphocyte attenuator was identified as another coinhibitory receptor of the Ig superfamily, related to CTLA-4 and PD-1 (174, 175). The highest BTLA level is detected in B cells but is also expressed on T cells, DCs, and macrophages (174, 175). In contrast to other coinhibitory receptors, BTLA is moderately expressed on naïve T cells, transiently upregulated upon antigenic stimulation, and downregulated in activated T cells (176). BTLA is highly expressed in anergic T cells generated *in vivo* (177). Although BTLA has a different expression pattern from other coinhibitory receptors, extensive studies have showed that BTLA has a direct inhibitory effect on T cell proliferation and cytokine production. *In vitro* stimulation of BTLA-deficient T cells resulted in enhanced proliferation in response to anti-CD3 (174, 175). Conversely, retroviral overexpression of BTLA in DO11.10 cells suppressed anti-CD3-mediated production of IL-2 (175). BTLA-deficient mice develop elevated autoantibodies and organ infiltration by activated CD4^+^ T cells indicating that BTLA has an active role in T cell tolerance (178). Consistent with a role of BTLA in maintaining peripheral tolerance, BTLA-deficient mice have increased susceptibility to experimental autoimmune encephalitis, whereas induction of oral tolerance is also compromised (175, 179).

A common ligand for CD160 and BTLA is the HVEM, which is a member of the tumor necrosis factor receptor superfamily, also known as TNFR superfamily 14 (180, 181). As mentioned above, the interaction of BTLA, which belongs to the Ig superfamily, with HVEM, which is a member of the TNF receptor superfamily, is a rare example of cross talk between members of different families of coinhibitory receptors and ligands. HVEM also interacts with another TNFR-associated factor-associated receptor, TNF receptor-like molecule 2, or LIGHT receptor (182). HVEM was initially discovered as a molecule that interacts with viral glycoprotein D to facilitate entry of herpes simplex virus (183). It is expressed in hematopoietic cells and nearly all parenchymal tissues with highest expression in lung, liver, and kidney, and lower expression in brain, pancreas, heart, placenta, and skeletal muscle (183). HVEM is expressed in high levels in naïve T cells, is transiently decreased after activation and is upregulated at the late activation stage (180, 184). Interaction of HVEM with LIGHT induces T cell proliferation whereas blockade of LIGHT: HVEM interaction inhibits responses of activated T cells (182). Thus, the CD160/BTLA/LIGHT/HVEM pathway mediates coinhibitory signals through the engagement of CD160 and BTLA with HVEM but promotes T cell activation by engagement of LIGHT. The distinct functions of HVEM ligands are mediated *via* distinct binding sites on HVEM. Specifically, HVEM has three cysteine-rich domains (CRDs) and a fourth CRD that is less typical and has only two of the three disulfide bonds (18, 183). The CRD1 is required for coinhibitory binding of HVEM with CD160 and BTLA but has no role in the costimulatory binding with LIGHT (180, 181). Deletion of CRD1 can convert the function of HVEM from a coinhibitory receptor, which seems to be its net effect, into a solely costimulatory receptor (181).

Little is known about the signaling pathways engaged by HVEM downstream of BTLA and CD160. BTLA contains two conserved ITIMs in its cytoplasmic tail (129), which are phosphorylated on Y274 and Y299 upon BTLA ligation and result in recruitment of SHP-1 and SHP-2 (18, 174, 175, 185). CD160, a GPI-anchor protein, is localized at the lipid rafts. By this localization, CD160 can regulate activation of key components of the TCR-proximal signaling machinery when engaged in cytolytic function (186). However, the mechanism *via* which CD160 inhibits T cell responses remains elusive. Currently, there is no information of how CD160 and BTLA might affect T cell immunometabolic programs. The involvement of BTLA in the maintenance of self-tolerance, the role of CD160 as coinhibitory receptor on T_EX_ cells, and their reciprocal expression in naïve versus activated T cells strongly suggest that together CD160 and BTLA must play an important role in the fine tuning of metabolic programs that determine T cell fate and function upon encounter of self-antigens, pathogens, or tumor antigens. Furthermore, the ability of HVEM to switch its properties from being a mediator of inhibitory signals *via* interaction with CD160 and BTLA to being a T cell costimulator *via* interaction with LIGHT indicates that the CD160/BTLA/LIGHT/HVEM pathway might regulate immunometabolic plasticity that rapidly switches T cell differentiation programs and guides distinct functional outcomes.

## T Cell-Intrinsic Immunometabolic Regulations Mediated by Checkpoint Inhibitors

The extensive studies regarding the expression and function of coinhibitory receptors provide currently emerging evidence that these receptors have synergistic roles in suppressing T cell function and provide evidence for their non-redundant roles in inhibiting the responses of activated T cells. Although the evolutional purpose for the coexpression of multiple coinhibitory receptors is not clearly understood, several observations provide challenging hypotheses. It is possible that coinhibitory receptors are expressed in distinct combinations on T cell subsets depending on the type of activation signals that such T cells receive, which might depend on the engagement of distinct costimulatory receptors or on the activation of autocrine and paracrine cytokine loops. For example, expression and recruitment of CTLA-4 to the immunological synapse depend on the strength of the TCR signal (107). It is also possible that ligands for the different coinhibitory receptors are differentially expressed in various tissues or tissue resident APC and, as a consequence, distinct coinhibitory receptors control T cell responses in distinct microenvironments. For example, parenchymal tissues and organs express ligands for PD-1 but not for CTLA-4 (20). As nutrients and metabolites provide important environmental cues with decisive role in T cell activation (187), it is also possible that distinct metabolic programs of various microenvironments selectively promote the expression and function of different coinhibitory receptors. For example, protection from oxidative damage mediated by reactive oxygen species (ROS) by an SOD-mimetic compound, resulted in elevated expression of LAG-3 in T cells and inhibition of TCR-mediated Th1 cell activation and effector function (139). Such metabolism-driven events might also differentially regulate the expression of coinhibitory receptors in T cell subsets (53).

Currently, there is little known about the metabolic repercussions of coinhibitory receptors on T cells and the metabolic consequences by targeting these immune checkpoint pathways in pathogen-specific and tumor-specific T cells. The immunometabolic effects mediated by the most clinically relevant coinhibitory receptors, CTLA-4 and PD-1, are starting to be elucidated. Laboratory studies have provided evidence that inhibitory checkpoint pathways can alter the metabolic program of T cells (126, 188). PD-1 engagement makes T cells unable to engage in glycolysis, glutaminolysis, or amino acid metabolism but increases FAO (126). This effect is due to inhibition of transport and utilization of glucose and glutamine. In contrast, PD-1 promotes FAO of endogenous lipids by inhibiting the lipid oxidation PI3K pathway, resulting in increased expression of carnitine palmitoyltransferase 1A (CPT1A), the rate-limiting enzyme of FAO, which plays an important role in the utilization of fatty acids as an energy source. In parallel, PD-1 abrogates the induction of fatty acid synthase and, as a consequence, decreases anabolic lipid metabolism and biosynthesis of lipids, which normally occurs during T cell activation.

Compared to T cells activated without PD-1 ligation, activated T cells receiving PD-1 signals have lower extracellular acidification rate (ECAR), an indicator of glycolysis, and lower oxygen consumption rate, an indicator of OXPHOS. These findings indicate that T cells receiving PD-1 signals are rather metabolically quiescent and preferentially use OXPHOS over glycolysis as indicated by the higher OCR/ECAR ratio. As fat-based metabolism is associated with longevity (189), the augmentation of FAO may explain the longevity of T cells, which engage the PD-1 pathway, in patients with chronic infections and cancer and their ability to be reinvigorated by blockade of PD-1. The role of PD-1:PD-L1 pathway in restricting glucose metabolism *in vivo* was also reported in a study of graft-versus-host disease in mice, where recipients of allogenic bone marrow deficient in PD-L1 had higher levels of Glut1 and lactate production (190).

Because of the increased FAO and the elevated OCR/ECAR ratio, it is possible that PD-1-mediated inhibitory effects in T cells might be related to a more oxidative environment. Although depletion of the antioxidant glutathione (GSH) was observed in T cells stimulated with or without PD-1 ligation, consistent with the expected increase of ROS during TCR-mediated activation (191, 192), PD-1 ligation resulted in more pronounced decrease in the levels of reduced GSH. However, under these conditions, T cells had higher levels of cysteine-GSH disulfide and ophthtalmate, a GSH-like product that is synthesized by the same enzymes (126). These changes might be suggestive of a more oxidative environment in T cells receiving TCR signals in conjunction with PD-1 ligation. However, several other tentative mechanisms might be implicated in these observations. In alloreactive T cells after allogeneic bone marrow transplantation, PD-1 expression has been correlated with an oxidative environment, ROS production, and higher susceptibility of these cells to metabolic inhibition by F1F0-ATP synthase complex inhibitors (193). Thus, expression and function of PD-1 and possibly other coinhibitory receptors might be linked with the T cell oxidative state, which can serve as a fine-tuning system of cellular differentiation.

A key mediator of oxidative detoxification is the PPARγ coactivator-1a (PGC-1α) (194, 195). Induction of PGC-1α during caloric restriction mediates selective cell adaptations that provide modifications permissive for cell survival and function under increased OXPHOS. Because PGC-1α is regulated by mTOR (196), which is downstream of PI3K/Akt pathway that is targeted by PD-1 (120, 121), it was hypothesized that by dysregulating PGC-1α expression and function, PD-1 might impair oxidative detoxification. Moreover, different subsets of T_EX_ cells might have distinct metabolic and bioenergetics properties such as mitochondrial biogenesis, which might correlate with glycolysis, SRC, CPT1A, and PGC-1α (126). Consistent with this hypothesis, studies in chronic LCMV infection, which leads to the generation of various subsets of T_EX_ cells, showed that the development of intermediate PD-1 (PD-1^int^) T_EX_ cells correlated with reduced glucose uptake and glycolysis, dysregulated mitochondrial bioenergetics, and suppressed PGC-1α expression. PGC-1α expression was enhanced in the absence of PD-1, and forced expression of PGC-1α partially reverted some of the metabolic derangements of PD-1^Int^—but not in PD-1^Hi^—T_EX_ cells and improved effector function (197). Similarly to the observations of *in vitro* stimulated human T cells (126), transcriptional analysis during T_EX_ development in response to LCMV infection revealed enrichment of genes involved in OXPHOS, particularly CPT1A (197). While these results point to a strong connection between PD-1-mediated metabolic reprogramming and induction of T cell exhaustion, it should be pointed out that similar bioenergetics mechanisms of T cell exhaustion *in vivo* mediated by chronic infection and cancer can also occur in a PD-1-independent manner (198, 199). Thus, other coinhibitory receptors might mediate similar immunometabolic effects, thereby resulting in differentiation of functionally impaired T cells.

As mentioned above, PD-1 together with several other coinhibitory receptors coregulate T cell exhaustion during cancer and chronic infections. Pathogen-specific CD8^+^ T cells in mouse models and humans with chronic viral infections as well as TILs from mouse tumor models and patients with cancer can coexpress multiple repressors, including PD-1, LAG-3, TIM-3, TIGIT, and others. The pattern and number of coinhibitory receptors expressed on the same cell can dramatically influence the severity of exhaustion (114, 115). T cell exhaustion is an active process and can lead to measurable consequences on T cell function. Studies have shown that distinct subsets of T_EX_ cells exist, which have different potentials for recovery after PD-1 blockade. Although T_EX_ cells with PD-1^int^ expression can be reinvigorated by PD-1 blockade, T_EX_ cells with high PD-1 (PD-1^high^) expression cannot (115). These distinct subsets of T_EX_ cells may also have different metabolic and bioenergetics properties depending on the nature of coinhibitory receptors expressed and engaged on their surface. Identification of the unique bioenergetics profiles of T_EX_ subsets might provide new tools to identify the level of exhaustion mediated by the combined and synergistic effects of coinhibitory receptors. It might also provide novel targets to reverse exhaustion and might serve as meaningful biomarkers to assess response to checkpoint inhibitor therapies.

In contrast to the effects of PD-1 in T cell metabolic reprogramming, CTLA-4 inhibits glycolysis without augmenting CPT1A and FAO, suggesting that CTLA-4 maintains immune quiescence by preserving the metabolic profile of non-stimulated cells (126). RNA-seq of TILs from progressor sarcoma tumors in mice that were either treated with control, anti-CTLA-4, anti-PD-1 blocking antibodies, or a combination of anti-CTLA-4 and anti-PD-1 antibodies revealed differential gene enrichment. PD-1 blocking showed enrichment predominantly in metabolic genes, while CTLA-4 blocking showed enrichment in cell cycle and effector memory genes. The combination of PD-1 and CTLA-4 antibody treatment revealed synergism of metabolic and T cell effector genes that correlated with the strongest enhancement of effector molecules (108). The distinct properties of CTLA-4 and PD-1 in affecting T cell metabolic reprogramming might be related to their differential ability to oppose signals mediated by the TCR. CTLA-4 mediates a potent inhibition of T cell activation, and CTLA-4 recruitment at the immune synapse is regulated by the strength of TCR signal (107). In contrast, PD-1 inhibits weak but not strong TCR signals (200). As a consequence, TCR-mediated biochemical events might be impaired but not fully abrogated (124, 201). Notably, the strength of PD-1 signaling also differentially affects T_EFF_ cell function (201). This rheostat effect of PD-1 on TCR signaling that likely depends on the level of PD-1 expression might mediate fine tuning of metabolic reprogramming leading to distinct T cell functional fates. The different immunometabolic implications of CTLA-4 and PD-1 in T cells, serve as a paradigm that provides evidence for the purpose of multiple coinhibitory receptors, which likely have unique roles in regulating immunometabolic programs and functional commitment of T cells. Mechanistic understanding of the immunometabolic effects of coinhibitory receptors will shed light not only to the state of T cell functional impairment and exhaustion in chronic infections and cancer but also to the metabolic regulation of T cell tolerance.

## Coinhibitory Receptors Affect Immunometabolic T Cell Responses by Regulating Cross Talk with Innate Immune Cells and Cancer

**Figure 4:**
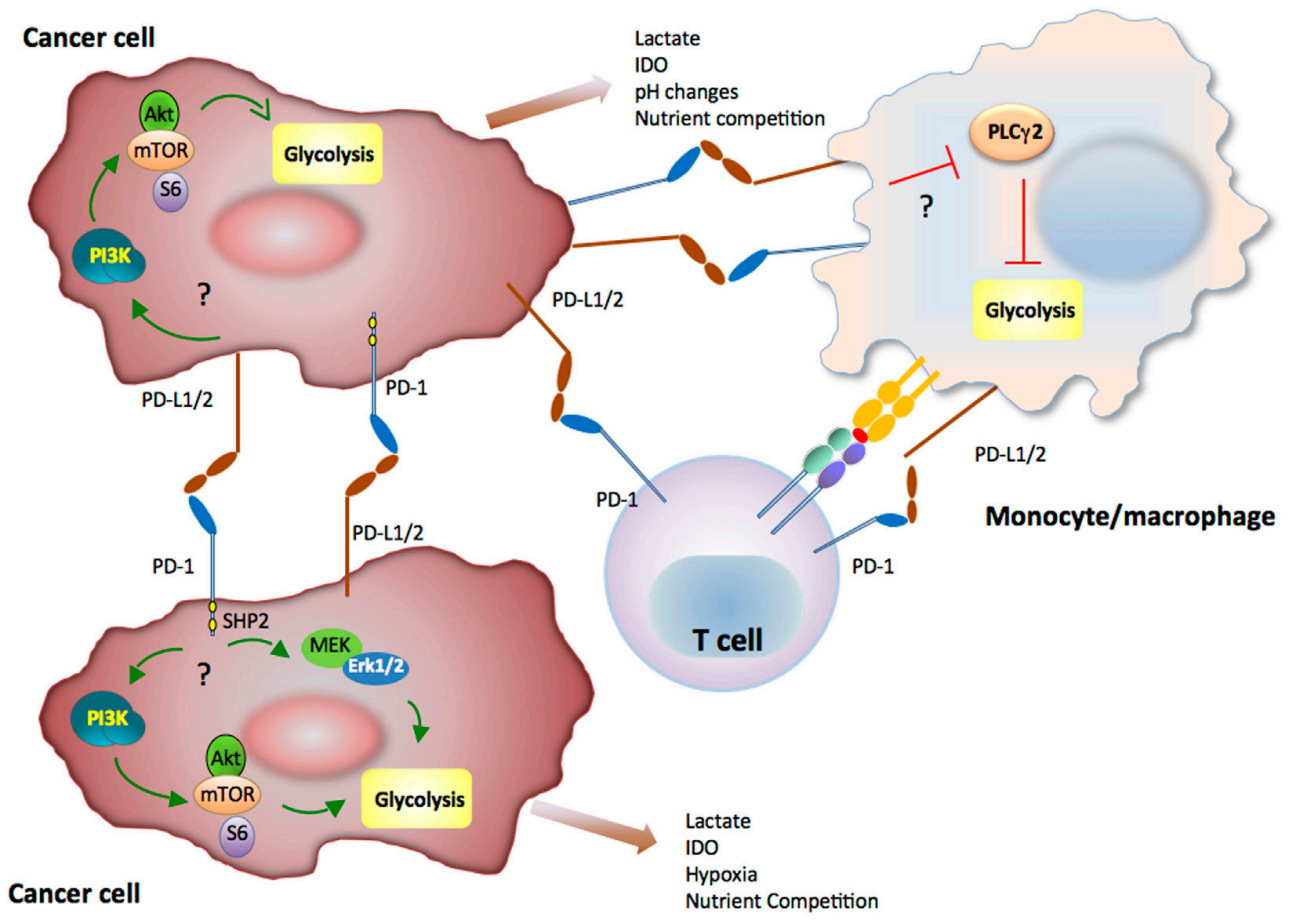
**Immunometabolic regulations mediated by coinhibitory receptors in innate immune cells and cancer can impact T cell immune responses**. Metabolic signaling mediated by the programmed death-1 (PD-1):programmed death-ligand 1 (PD-L1) pathway in cancer or myeloid cells of the tumor microenvironment might subsequently alter T cell metabolic reprogramming and immune function due to nutrient competition and by generating metabolic products with detrimental effects on T cell immune function. PD-1 and PD-L1 might activate glycolysis in cancer cells, whereas PD-1 ligation might inhibit glycolysis of monocytes/macrophages in the context of cancer.

It has become increasingly understood that coordinated metabolic switches in T cells and innate immune cells modulate cellular activities and contribute to the progression of cancer and chronic infections. Metabolic communication among T cells, innate immune cells, and cancer might contribute to immunometabolic regulations and fine tuning of the activation and antitumor responses of T cells and myeloid cells (202) (Figure 4). In this regard, immunometabolic regulations mediated by coinhibitory receptors can impact T cell immune responses due to direct T cell-intrinsic signaling and by altering the metabolic properties and the differentiation program of innate immune cells (203). Not only the coinhibitory receptor ligands but also the coinhibitory receptors themselves are also present in various types of innate immune cells in addition to T cells. Intriguingly, it was recently reported that the PD-1: PD-L1 axis is implicated in immune metabolic dysfunctions of monocytes in chronic lymphocytic leukemia (203). In that system, triggering PD-1 checkpoint on monocytes hampers glycolysis and phagocytosis. Conversely, disrupting PD-1: PD-L1 signaling reverses these immune metabolic dysfunctions. Taken together, these findings imply a novel metabolic interplay between cancer cells and monocytes and that blocking PD-1: PD-L1 might restore metabolic together with antitumor activity of monocytes/macrophages.

In a sarcoma tumor model, it was reported that expression of PD-L1 on cancer cells was associated with cell-intrinsic signaling *via* the PI3K/Akt pathway and mTOR, leading to expression of glycolysis genes and enhanced glycolytic metabolism (204). Although it is unclear whether PD-L1 can trigger reverse signals to tumor cells by its short cytoplasmic tail that does not have obvious signaling motifs, PD-L1 functions as an inhibitory receptor that protects cancer cells from immune-mediated cancer cell destruction and Fas-mediated killing (205). Because cancer cells have elevated activation of the PI3K/Akt pathway and are highly glycolytic, expression of PD-L1 might result in concomitant increase of PI3K/Akt activation and elevated rate of tumor-intrinsic glycolysis as a consequence of improved survival. It has also been observed that subpopulations of certain human and murine melanoma cell lines and subpopulations of malignant cells from patients with melanomas express PD-1 (206, 207). However, in contrast to T cells, in which PD-1 ligation inhibits the activation of PI3K/Akt, MAPK, and mTOR pathways, ligation of PD-1 in melanoma cells was reported to activate these pathways and to promote expression of glycolytic enzymes, which correlate with tumor growth (206). To date, such finding of cancer cell-intrinsic growth-promoting effects of PD-1 have not been validated in tumor models and primary cancer cells. It is also unclear whether PD-L1 naturally expressed on tumor cells can trigger activation of glycolytic metabolism in cancer cells and whether such effect might be associated with tumor growth. However, together these findings (203, 204, 206) suggest that cell-intrinsic metabolic signaling mediated by the PD-1:PD-L1 pathway in cancer or myeloid cells of the tumor microenvironment might subsequently alter T cell immunometabolism and immune function due to nutrient competition and by generating metabolic products with detrimental effects on T cell immune function (208).

## Concluding Remarks

Coinhibitory receptors have a major impact on the metabolic programs that drive the differentiation and functional fate of naïve T cells. These coinhibitory receptors have a major role in reprogramming the metabolic preferences of T_EFF_ cells leading to metabolic and functional switches. Because coinhibitory receptors mediate distinct effects on the activation of signaling pathways, the role of coinhibitory receptors on altering T cell immunometabolism is also anticipated to be distinct. Identifying and targeting the specific immunometabolic determinants relayed by different coinhibitory receptors might have significant clinical implications as selective receptor targeting might be employed in order to achieve specific desired modifications in the metabolic programs and functional fates of T cells.

Metabolic determinants of different microenvironments provide important cues to drive differentiation and function of T cells by metabolism-based mechanisms. Expression and engagement of coinhibitory receptors after T cell activation can provide the necessary signals to terminate immune responses by altering the metabolic programs of antigen-specific T_EFF_ cells. Such metabolic reprogramming might lead to conversion of T_EFF_ to T_REG_ and T_M_ for termination of immune activation and maintenance of the T cell repertoire, respectively. Understanding and recapitulating the metabolic programs mediated by coinhibitory receptors will have implications for the induction of self-tolerance and the treatment of autoimmune diseases. Conversely, blocking and reversing metabolic programs mediated by coinhibitory receptors will promote T_EFF_ differentiation and function and will improve immune-based therapies mediated by checkpoint inhibitors and by adoptive immunotherapy of *ex vivo* engineered T cells for the treatment of cancer and chronic infections. Exploiting the role of the coinhibitory receptors in T cells, innate immune cells and cancer will be a major challenge.

## Author Contributions

NP and JW wrote the main body of the manuscript and generated figures; LS wrote several sections of the manuscript; CH participated in the preparation of the manuscript and generated the Supplementary Material; PS participated in the writing of various sections of the manuscript; and VB had the overall supervision in the preparation of the manuscript and figures.

## Conflict of Interest Statement

Vassiliki A. Boussiotis has patents on the PD-1 pathway licensed by Bristol-Myers Squibb, Roche, Merck, EMD-Serono, Boehringer Ingelheim, AstraZeneca, Novartis and Dako. The authors declare no additional potential conflicts of interest.
