# Supplementary material for: Immunometabolic Regulations Mediated by Coinhibitory Receptors and Their Impact on T Cell Immune Responses
Source: Front Immunol. 2017 Apr 11;8:330. doi: 10.3389/fimmu.2017.00330 (PMC5387055; doi:10.3389/fimmu.2017.00330)
Supplement: Supplementary file 1 [file Table_1.docx]

**Glossary of Abbreviations**

ADAM - A disintegrin and metalloproteinase

ADP - Adenosine diphosphate

AMP - Adenosine monophosphate

AMPK - AMP-activated protein kinase

AP-1 - Adaptin1, Activator protein 1

APC - Antigen-presenting cells

ATP - Adenosine triphosphate

Bat3 - HLA-B associated transcript 3

BTNL2 - Butyrophilin-like protein 2

BTLA - B and T lymphocyte attenuator

CEACAM-1 - Carcinoembryonic antigen-related cell adhesion molecule 1

CK2 - Casein kinase 2

CTLA-4 - Cytotoxic T lymphocyte antigen-4

Con-A - Concanavalin A

CPT1A - Carnitine palmitoyltransferase 1A

DAG - Diacylglycerol

DC - Dendritic cell

DNAM-1 - DNAX accessory molecule-1

ECAR - Extracellular acidification rate

Eomes - Eomesodermin

Erk - Extracellular signal–regulated kinases

FAO - Fatty acid oxidation

FAS - Fatty acid synthesis

FASN - Fatty acid synthase

FOXP3 - Forkhead-Box P3

GAPDH - Glyceraldehyde phosphate dehydrogenase

GM-CSF - Granulocyte-macrophage colony-stimulating factor

GSH - Glutathione

Glut1 - Glucose transporter 1

HERV - Human endogenous retroviruses

HIF-1α - Hypoxia-inducible factor 1α

HHLA2 - HERV-H LTR-Associating 2

HIV - Human immunodeficiency virus

HK2 - Hexokinase 2

HLA - Human leukocyte antigen

HMGB1 - High mobility group box 1

HVEM - Herpesvirus entry mediator

IDO - Indoleamine-pyrrole 2,3-dioxygenase

IFNγ - Interferon γ

IL-2/7/12 - Interleukin 2/7/12

ITIM - Immunoreceptor tyrosine-based inhibition motif

ITK - IL-2 inducible T cell kinase,

IRF9 - Interferon regulatory factor 9

ITT - Immunoglobulin tail tyrosine

JAK - Janus kinase

LAG-3 - Lymphocyte activation gene 3

LCMV - lymphocytic choriomeningitis virus

LIGHT - Lymphotoxin-like, exhibits inducible expression, and competes with herpes simplex virus glycoprotein D for HVEM, a receptor expressed by T lymphocytes LKB1 - Liver kinase B1

LTR - Long terminal repeat

MAPK - Mitogen-activated protein kinase

MEK - MAPK/ERK Kinase

MHC - Major histocompatibility complex

mTOR - Mechanistic/mammalian target of rapamycin

NFAT - Nuclear factor of activated T cells

NF-kB - Nuclear factor 'kappa-light-chain-enhancer' of activated B-cells)

NK - Natural killer cells

NKT - Natural killer T cells

NY-ESO-1 - New York esophageal squamous cell carcinoma-1

OCR - Oxygen consumption rate

OXPHOS - Oxidative phosphorylation

SHC - Src Homology 2 domain-containing-transforming protein C1

PD-1 - Programmed death-1

PD-L1/2 - Programmed death-ligand 1/2

PEP - Phosphoenolpyruvate

PFK2 - Phosphofructokinase 2

PGC-1a - PPARγ coactivator-1a

PI3K - Phosphatidylinositol-4,5-bisphosphate 3-kinase

PI(3,4,5)P3 - Phosphatidylinositole 3,4,5-trisphosphate

PKM1/2 - Pyruvate kinase M1/2

PLC - Phosphoinositide phospholipase C

PP2A - Protein phosphatase 2

PPARγ - Peroxisome proliferator-activated receptor γ

PtdSer - Phosphatidyl serine

PTEN - Phosphatase and tensin homologue deleted on chromosome 10

Raptor - Regulatory-associated protein of mTOR

Ras-GRP1 - RAS guanyl-releasing protein 1

Rheb - Ras homolog enriched in brain

Rictor - Rapamycin-insensitive companion of mTOR

Rlk - Receptor-like kinase

RORγt – retinoic acid receptor related orphan receptor C

ROS - reactive oxygen species

S6K - Ribosomal protein S6 kinase

SHP-2 - Src homology-2 (SH2) domain-containing phosphatase

Snat1/2 - Sodium-coupled neutral amino acid transporter 1

sLAG-3 - Soluble form of LAG-3

STAT - Signal transducer and activator of transcription

SOD - Superoxide dismutase

SRC - Spare respiratory capacity

Tbx21 - T-box transcription factor 21

TCA cycle - Tricarboxylic acid cycle

TCR - T cell receptor

T_EFF_ - Effector T cells

T_EX_ - Exhausted T cells

TFAM - Mitochondrial transcription factor A

Th - T helper cells

TIGIT - T vell immunoglobulin and ITIM domain

TIL - Tumor-infiltrating lymphocytes

TIM-3 - T cell–immunoglobulin–mucin domain 3

T_M_ - Memory T cells

T_N_ - Naïve T cells

TNF - Tumor necrosis factor

TORC1/2 - mTOR complex 1/2

T_REG_ - Regulatory T cells

TSC - Tuberous sclerosis

VEGF - Vascular endothelial growth factor

ZAP70 - Zeta chain of T cell receptor associated protein kinase 70
